# Clinical Features and Laboratory Findings of Travelers Returning to South Australia with Dengue Virus Infection

**DOI:** 10.3390/tropicalmed3010006

**Published:** 2018-01-07

**Authors:** Emma J. Quinn, Allena H.-C. Cheong, Julie K. Calvert, Geoffrey Higgins, Trish Hahesy, David L. Gordon, Jillian M. Carr

**Affiliations:** 1 Microbiology and Infectious Diseases, College of Medicine and Public Health, Flinders University, Adelaide SA 5042, Australia; ejquinn377@gmail.com or emma.quinn@flinders.edu.au (E.J.Q.); allena.cheong@flinders.edu.au (A.H.-C.C.); Julie.calvert@flinders.edu.au (J.K.C.); d.gordon@flinders.edu.au (D.L.G.); 2 Infectious Diseases Laboratories SA Pathology, Adelaide, SA 5000, Australia; geoffrey.higgins@sa.gov.au (G.H.); trish.hahesy@sa.gov.au (T.H.)

**Keywords:** dengue virus, returned traveller, dengue serology, dengue RT-PCR

## Abstract

Reported cases of dengue are rising in South Australia (SA) in travellers returning from dengue-endemic regions. We have undertaken a retrospective analysis to identify the clinical and laboratory characteristics of patients returning to SA with suspected dengue virus (DENV) infection. From 488 requests, 49 (10%) were defined by serology as acute dengue, with the majority of patients (75%) testing as non-structural protein 1 (NS1) and/or IgM positive. Dengue was most commonly acquired in Indonesia (42.9%) with clinical features of fever (95%), headache (41%) and myalgia/arthralgia (56%). The presence of rash (36%) and laboratory findings of neutropenia, leukopenia, thrombocytopenia, but not elevated C-reactive protein, were distinct from findings in DENV-seronegative patients. Available dengue seropositive samples were analysed by RT-PCR, with 14/32 (43.8%) positive by a serotype non-specific DENV assay, but 28/32 positive (87.5%) when also assessed by serotype-specific RT-PCR. Serotype analysis revealed the predominance of DENV-1 and DENV-2 and the presence of DENV-3, but not DENV-4 or Zika virus (ZIKV). Thus, dengue in returned travellers in SA presents in a manner consistent with World Health Organization (WHO) definitions, with symptoms, travel history and laboratory results useful in prioritising the likelihood of dengue. This definition will assist the future management in DENV-non-endemic regions, such as SA.

## 1. Introduction

Dengue is a mosquito-borne disease caused by four serotypes of dengue virus (DENV-1, DENV-2, DENV-3 and DENV-4), belonging to the *Flavivirus* genus. The World Health Organization (WHO) identifies dengue as the most rapidly spreading arboviral disease in the world, and estimates that 50–100 million DENV infections occur each year [1], with mathematical modelling suggesting an even higher incidence [2]. The incidence of dengue has increased thirtyfold in the past 50 years, and the approximately 75% of the global population living within the Asia-Pacific region are potentially exposed to DENV [3,4]. The increase in global DENV incidence has been reflected in the trends in diagnosis of infections in travellers returning to Australia over the past decade [5]. In South Australia (SA), dengue notifications have increased from 50 in 2012 to 116 in 2016 (http://www.sahealth.sa.gov.au/). The increased incidence of dengue in Australia, in the context of increased international travel to dengue-endemic areas, has made it essential for Australian doctors to be able to recognise and manage dengue, and differentiate DENV infection from other mosquito-borne travel-associated diseases with similar clinical presentations including malaria, yellow fever, chikungunya and more recently, Zika virus (ZIKV) [6,7].

Dengue is transmitted through the bite of infected female mosquitoes of certain *Aedes* species. By far the most common of these vectors is *Aedes aegypti*, a mosquito found primarily in urban tropical and subtropical areas globally, and currently confined to parts of northern Australia [8]. The clinical manifestations of DENV infection range from asymptomatic to mild self-limiting febrile illness or to serious and life-threatening cases involving plasma leakage with or without haemorrhage [4,9,10]. After an incubation period of 3–10 days, the illness follows three phases in symptomatic patients: febrile, critical, and recovery [11,12]. During the acute febrile phase, which lasts 2–7 days, patients tend to present with at least one of either high fever, headache, retro-orbital pain, myalgia, arthralgia, rash, nausea or vomiting. These symptoms are non-specific and at this phase of the disease it is difficult to distinguish dengue from non-dengue febrile illnesses. In the full blood examination (FBE), a progressive drop in the total white cell count (WCC) is the earliest abnormality seen during the acute febrile phase [3,11]. A small number of patients progress to the critical phase, which occurs during a 24–48 h period around defervescence. During this phase, symptoms of plasma leakage, coagulation derangement or organ impairment may be observed. Severe dengue also includes patients with hepatitis, neurological disorders, myocarditis or severe bleeding without plasma leakage or shock. FBE features at this stage include an increase in haematocrit and a decrease in platelet count [4,11,13].

WHO released a revised case classification in 2009, transitioning from the old definitions of dengue fever, dengue haemorrhagic fever or dengue shock syndrome, to dengue with or without warning signs, and severe dengue. Warning signs under this classification system include abdominal pain, persistent vomiting, fluid accumulation, mucosal bleeding, lethargy, hepatomegaly >2 cm and an increase in haematocrit accompanied by a rapid decrease in platelet count. Severe dengue must include at least one of the following features: (a) plasma leakage; (b) severe bleeding, and/or; (c) severe organ impairment [11]. These guidelines aim to increase early recognition of warning signs and improve clinical triage and management decisions. Severe disease is seen in dengue-endemic areas and epidemiological observations suggest this is mostly associated with a secondary DENV infection of a different serotype to that of the initial infection [9,10,14]. Since travellers from DENV-non-endemic countries are usually DENV naïve, DENV cases in returned travellers would not be expected to develop severe disease. The multicultural nature of the Australian population who may not be DENV naïve, and increased multiple travels to neighbouring DENV-endemic countries, however, make this less of a certainty.

Laboratory testing is particularly important in the confirmation of dengue diagnosis because infection produces such a wide range of symptoms, many of which are non-specific. Techniques for laboratory diagnosis of DENV infection involve direct detection of viral nucleic acid (by RT-PCR) or antigens (such as NS1) or serological tests for detection of IgG/M antibodies, and frequently involves a combination of these approaches [3,4,11,12]. SA Pathology, the SA government pathology service and diagnostic laboratories, is the only place for DENV testing in SA, where a rapid immunochromatographic test for IgM, IgG and NS1 antigen is performed. This test is chosen due to the low volume of dengue requests and a need for rapid turn-around of results to clinicians. The value of a nucleic acid testing approach in this setting has not been evaluated. In addition to specific diagnostic tests to demonstrate DENV infection, FBE in particular for WCC, platelets and haematocrit, are of particular importance to align with clinical symptoms to diagnose dengue and determine severity of disease.

Here we defined the characteristics of the patient cohort, circulating strains and the diagnostic process for DENV in SA to assess the need for additional routine diagnostic tests and, to define clinical parameters useful for management of DENV in this setting where the disease is rarely seen, but is likely to be of growing importance.

## 2. Materials and Methods 

### 2.1. Sample Collection 

This retrospective descriptive study includes 488 dengue tests performed over a 13-month period between 1 January 2014 and 31 January 2015. Samples were identified from SA Pathology DENV diagnostic worklists and database records searched to identify test requests and results from the same clinical episode. Sera was obtained from archival material remaining after completion of diagnostic testing and stored at −20 °C. Demographic data, travel history and clinical presentations were obtained from diagnostic request forms or hospital discharge summaries. All patient data collected were de-identified and stored securely to maintain privacy, in accordance with the Southern Adelaide Clinical Human Research Ethics Committee (SACHREC), approval number 200/15. Stored patient sera were obtained under SACHREC approval number 134/15, with all undertakings in accordance with the World Medical Association Declaration of Helsinki.

### 2.2. Serological Testing for DENV 

Serological testing was performed on sera or plasma by Dengue Duo IgM/IgG/NS1 assay (SD BIOLINE Dengue Duo, Alere) in accordance with the manufacturer’s instructions.

### 2.3. RNA Extraction and pan-DENV RT-PCR 

RNA was extracted from 200 µL of sera using the High Pure Viral Nucleic Acid Kit (Roche Applied Sciences) and resuspended in 30 µL volumes. Stored sera were not available for all DENV-seropositive samples and RT-PCR was performed on the available DENV-seropositive samples (*n* = 32) and randomly selected DENV-seronegative samples (*n* = 8) as negative controls. A one-step pan-DENV real-time RT-PCR was performed using SuperScript III Platinum One-Step qRT-PCR kit (Invitrogen), with primers as developed by Warrilow et al. [15] with fluorescent probe detection (Table 1). Two µL of RNA was added to a 10 µL reaction with 1 μM forward and reverse primers, 0.25 µL of SuperScript III Reverse Transcriptase/Platinum Taq mix, 0.05 μM probe. Thermal cycling was performed with reverse transcription at 50 °C, 15 min; activation of Taq DNA polymerase at 95 °C, 10 min; 45 cycles at 95 °C, 10 s; 55 °C, 15 s and 60 °C, 30 s. Amplification and detection was performed in a Rotor-Gene 6000 (Qiagen) and expected PCR products confirmed by agarose gel electrophoresis. All experiments included an internal positive (3000 or 10^4^ genome copies per μL) DENV RNA control and a standard curve of DENV-2 MON601 DNA [16] from 15–0.015 pg/μL. 

### 2.4. Serotype-Specific DENV RT-PCR and ZIKV RT-PCR 

Serotype-specific DENV or ZIKV RT-PCR was performed on RNA extracted as above. In brief, 5 µL of RNA was annealed with 2 µM random hexamers (NEB), 10 mM dNTPs in a 14 µL reaction by denaturation at 65 °C, 5 min and rapid cooling to 4 °C. 100 U SuperScript IV Reverse Transcriptase, 5 mM DTT and 15 U RNase Inhibitor was added in a final reaction of 20 µL and incubated at 23 °C, 10 min, 55 °C, 10 min, 80 °C 10 min and cooled to 4 °C. One-tenth of the cDNA reaction was diluted a further 1/10 *v*/*v* and amplified in a 10 µL reaction with iTaq sybergreen mix (Biorad) and 10 µM of primers, as described in Table 1. Reactions were cycled in a Rotor-Gene 6000. Serotype positivity was determined by a positive real-time RT-PCR result and confirmation of the PCR product by agarose gel electrophoresis. All experiments included positive controls ranging from 50–0.005 pg/µL that consisted of the cloned target regions of the DENV genome from DENV-1 (GenBank accession #U88535, nucleotides [nt] 8603-8709), -3 (GenBank accession #M93130, nt 118–241) and -4 (GenBank accession #M14931, nt 187–293) strains, DENV-2 Mon601 DNA, as above or ZIKV RNA extracted from PRVABC49 strain. Additionally, DENV-2 RNA extracted from MON601 infectious virions were used as a control to define the cross-reactivity of the serotype specific RT-PCRs. 

### 2.5. Data Analysis 

Categorical variables are presented as counts and proportions, and continuous variables are presented with medians and interquartile ranges. Data were analysed in GraphPad Prism v 6.07 with significance determined using Fishers exact test and Student’s unpaired *t*-test, with Welch’s correction.

## 3. Results

### 3.1. Dengue Positive Samples Represent a Minority of DENV Test Requests and Are Mostly NS1/IgM Positive 

From 488 requests for DENV diagnostic testing processed between 1 January 2014 and 31 January 2015, 55 tests returned a positive result for at least one of IgG, IgM or NS1 (Table 2). Only five test sets (10%) were ordered as convalescence follow-up and convalescence sera were excluded from our analysis. A single patient with no acute symptoms of dengue returned a positive test for DENV IgG only, on the background of a known previous DENV infection, and was excluded from the DENV-seropositive group. Thus, there were 49 unique patients with a positive acute DENV result representing 10% of DENV requests and 433 patients for whom DENV testing was requested but returned serological negative results. Of the five patients that underwent follow-up for seroconversion, all were DENV IgM-positive at follow-up, but only a single patient demonstrated seroconversion to IgG with the timing of follow-up testing between seven and 22 days after the initial DENV test. DENV RT-PCR-positive samples were found across the breadth of the serological responses (Table 2), and RT-PCR results are discussed in more depth in following sections.

### 3.2. Demographics of the Patient Cohort with Clinical Dengue Serology Requests 

Of the 433 patients who tested negative for DENV, 51 were randomly selected for comparison to the DENV-seropositive cohort that satisfied both (a) the availability of clinical and laboratory data; and (b) selection of patient samples in each month across the study time frame. The demographic information is indicated in Table 3, with comparable age and gender distribution between the DENV-seropositive and -seronegative cohorts. DENV-seropositive patients arose equally from requests generated from hospital and community centres of care.

Since dengue is not locally transmitted or acquired in SA, the travel history of this cohort was assessed (Table 4). The most common travel destination in both the DENV-seropositive and seronegative groups was Southeast Asia (SEA), with Indonesia the most frequent destination. Interestingly, two patients in the DENV-seronegative cohort explicitly recorded no recent overseas or interstate travel, with one of these tests requested from a major public hospital. Notably, three requests were associated with travel history to North Queensland, reflecting the knowledge of clinical requestors of the risk of DENV infection associated with this within-Australia travel destination.

### 3.3. Clinical Presentations and Laboratory Measurements in the Patient Cohort with Clinical DENV Serology Requests 

Fever was the most common symptom reported by all patients for whom DENV testing was requested, and was significantly more frequent in patients positive for acute DENV infection than those seronegative for DENV (Table 5). Additionally, there were significantly more patients in the DENV-seropositive cohort who presented with a rash (Table 5). Dengue warning signs were noted in 7 (14%) of the DENV-seropositive patients, with abdominal pain; 2 (4%), persistent vomiting; 4 (8%), lethargy; 5 (10%), and mucosal bleeding in 1 (2%) but no patients were recorded to develop severe dengue. A specific diagnosis was recorded in 35/51 (68.6%) of the DENV-seronegative patients, which could be grouped as parasitic, respiratory or enteric causes, but also included a variety of other infectious and non-infectious aetiologies (Table 6). 

**Figure 1:**
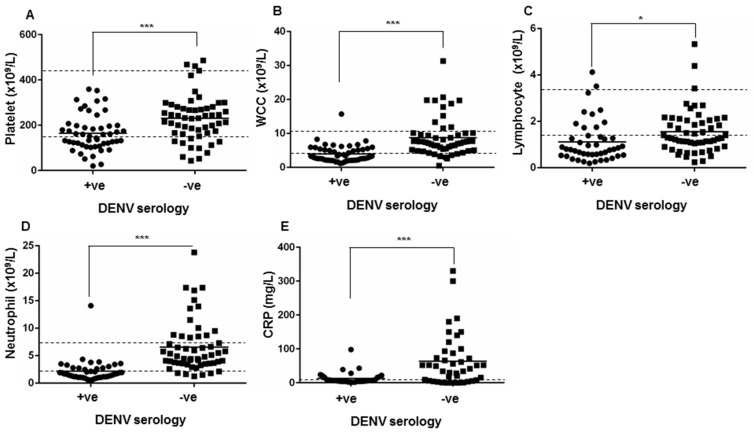
Comparison of (**A**) platelet count; (**B**) white cell count (WCC); (**C**) lymphocyte count; (**D**) neutrophil count and (**E**) C-reactive protein (CRP) levels; in blood from patients who were positive (+ve, *n* = 49) or negative (−ve, *n* = 51) by DENV serology. * = significantly different, *p* < 0.05; *** *p* < 0.0005, Students *t*-test. Clinical reference range is indicated by the dashed lines.

Analysis of the blood profile of DENV-seropositive patients demonstrated low median platelet, WCC, lymphocyte and neutrophil counts (Table 7). The median serum level of C-reactive protein (CRP), a commonly-used inflammatory marker, was within the normal range in DENV-seropositive patients. Comparison of laboratory measures in the blood of DENV-seropositive and -seronegative patients revealed significant differences in multiple parameters. While the median value in the DENV-seronegative group suggested lymphopenia, lymphocyte levels were significantly lower in the DENV-seropositive group (Table 7). In contrast, the DENV-seronegative group had clinically normal platelet, WCC and neutrophil levels with measurements significantly higher than those seen in the DENV-seropositive group (Table 7). The primary profile from the FBE demonstrated clinical thrombocytopenia (24/49; 49.0%), leukopenia (30/49; 61.2%), lymphopenia (34/49; 69.4%) and neutropenia (25/49; 51.0%) in a large proportion of the DENV-seropositive patient cohort (Figure 1). In contrast, the primary clinical FBE profile in the DENV-seronegative group showed elevated CRP (>50 mg/L in 18/51; 35.3%), and neutrophilia in 19/51 (37.3%) of patients (Figure 1).

### 3.4. Reliable Detection of DENV by RT-PCR Requires Multiple PCR Approaches and Detects DENV-1, -2 and -3 Serotypes 

Serologically DENV-positive samples were also analysed by using a serotype non-specific pan DENV RT-PCR, as well as separate serotype-specific RT-PCR assays for DENV-1, -2, -3 and -4. Only 14/32 samples (43.8%) were RT-PCR positive by pan-DENV RT-PCR, which increased to 28/32 (87.5%) when serotype specific RT-PCRs were additionally employed (Table 2). This highlights a lower sensitivity or potential inability of the pan-DENV RT-PCR to detect many DENV strains in this cohort. Of the 14 pan-DENV positive RT-PCR samples, four could not be serotyped, one was DENV-1, four were DENV-2 and five were mixed DENV-1/-2 positive (Table 8). Serotype specific RT-PCRs identified 17 samples with a clear single serotype, 7 with mixed serotype and 8 samples that could not be serotyped. Specifically, results demonstrated 11 DENV-2, five DENV-1 and one DENV-3 strains. Mixed RT-PCR positive results were observed with DENV-1/-2 (*n* = 5) and DENV-2/-3 (*n* = 2) (Table 8). No DENV-4 positive samples were identified. Additionally, samples were screened with a ZIKV RT-PCR but no positive samples were identified.

## 4. Discussion

Dengue in Australian travellers is an important differential diagnosis to consider in febrile patients returning from Northern Queensland or overseas from DENV-endemic regions. While there have been a number of retrospective studies in non-endemic countries [19,20,21,22], this is the first study of DENV infection in returned travellers in an entire presenting cohort in one Australian state that represents the breadth of the clinical population in the hospital and community setting, returning both DENV-seropositive and DENV-seronegative results. 

In this setting, 10% of the clinical dengue requests returned a DENV-positive serological result, suggesting that DENV infection is a significant but not major cause of fever in the returned traveller in SA. This is consistent with other studies, which suggested an incidence of dengue in the returned traveller of 8% in Canada, 2014–2015 [23]; 6.9% in Boston, USA, 2008–2009 [24]; 8.7% in Belgium, 2010–2013 and 2.7% across Europe [25]. The majority of our dengue cases present with positive NS1 serology with or without positive serology for IgM. Since NS1 is reported to be present from as early as day 1 and up to day 9 post-infection [26], this represents patients seeking clinical assistance in the early stages of DENV infection. Very few patients underwent follow-up to assess seroconversion (10%) and while the outcome confirmed the presence or seroconversion to IgM, only one patient seroconverted to detectable IgG, 2–3 weeks after acute illness. This seems quite late compared to prior reports of DENV IgG detectable by ELISA 11 days following primary illness [27] and may reflect a lower sensitivity or high level of false positives for detection using the DENV immunochromographic test. Given the good correlation of the DENV immunochromographic test with RT-PCR results, the latter is unlikely.

The majority of the patients in this study acquired DENV during travel to SEA, with Indonesia and Thailand the most common travel destination within this area. Similarly, from 208 patients in three hospitals in Melbourne and Darwin, 94 patients (45%) acquired DENV following travel to Indonesia and 19% from Thailand [20], while SEA was identified as an important source of DENV infection in the returned traveller in Queensland [28]. This is also consistent with the ‘2016 yellow book maps’ that suggest Indonesia as a ‘frequent and continuous source’ of DENV [29]. Again, studies of travellers from Belgium and Hamburg have shown that the main travel destination where DENV is acquired is SEA, although in contrast to Australia, the specific country of travel acquisition was predominantly Thailand [21,30]. 

In terms of risk of acquisition, a prospective study in Melbourne-based travel clinics assessed the incidence and seroprevalence of DENV infections specifically in travellers returning from Asia [31]. This study reported an estimated incidence of 3.4 infections per 10,000 traveller days. Rocklov et al. report in a study of 925 Swedish travellers from 1995–2010, an ‘attack-rate’ for DENV of 13.6/100,000 travellers [32]. This attack-rate, however, increased to 40–50 for travellers to Sri Lanka, Bangladesh or Thailand, highlighting the greater risk for these travel destinations, that are also part of the SA travellers’ itineraries. 

The demographics of potential dengue patients in SA were as expected, with a 30–40-year-old median age and a relatively even gender distribution, likely reflecting the demographics attracted to travel to SEA. Dengue clinical requests and positive results were evenly distributed between the community and hospital setting, reflecting the predominance of mild DENV disease in this cohort. The majority of patients in both the DENV-seropositive and -seronegative groups presented with a febrile illness. The FBE performed at presentation revealed that those patients who were subsequently found to have DENV tended to present with a classical picture of leukopenia and thrombocytopenia, with lymphopenia and neutropenia also observed. DENV-seropositive patients were also found to have comparatively low CRP values. Low CRP in DENV patients has been previously documented with only 4/119 patients having a CRP >50 mg/L, although the same study reported a mild elevation of >5 mg/L CRP in 25% of patients with dengue fever [21]. Similarly, elevated CRP has been reported in comparison to healthy controls, although CRP levels were still generally below 10 mg/mL [33]. In contrast, those patients who were subsequently found to be DENV seronegative did not tend to present with leukopenia or thrombocytopenia, and had higher neutrophil and CRP values. These DENV-seronegative febrile returned travellers were diagnosed with a variety of expected travel-associated conditions such as respiratory, gastrointestinal and parasitic infections. Additionally, the presence of rash, seen in 36% of DENV-seropositive patients was higher compared to the DENV-seronegative group. In future practice, it may be useful for clinicians to recognise that a combination of low CRP, thrombocytopenia, neutropenia and leukocytopenia with rash makes DENV a likely clinical diagnosis in the febrile returned traveller. This could improve management in terms of prescription of empiric antibiotic therapy, and the avoidance of administration of potentially harmful non-steroidal anti-inflammatory agents. The latter were reportedly administered in 22% of hospitalised DENV-infected patients [20] but were not recorded as being prescribed in our study cohort.

There have been a number of similar retrospective studies of dengue clinical presentations in non-endemic countries [19,20,21,22]. These studies used the revised dengue classifications outlined by the WHO in 2009 [11] to determine the number of returning travellers who met the criteria for dengue with or without warning signs, and severe dengue. Recently, the largest of these Australian trials was conducted across four healthcare networks in Victoria and the Northern Territory [20]. This study, over the period 2012 to 2015, found that 40% of hospitalised patients met the criteria for dengue with warning signs, and one met the criteria for severe dengue [20]. This is higher than seen in our cohort, where 14% of patients presented dengue warning signs and no patients developed severe dengue. Our study cohort largely represents DENV-naïve travellers, with only 6/49 (12.2%) positive for IgG and thus potential secondary DENV infection. Since secondary infections are more likely to be associated with severe dengue, it is not surprising that our study cohort did not include any cases of severe dengue. In contrast, the study of Tai et al. reported 22% of hospitalised DENV cases with serological evidence of prior DENV exposure [20]. This higher incidence of secondary DENV may reflect differences in the travel history between the Adelaide and Melbourne/Darwin cohorts, the higher number of patients in the study of Tai et al. [20], or the bias in selection of hospitalised DENV cases, compared to our Adelaide cohort that reflected all clinical DENV cases in both community and hospitalised settings.

Importantly, while only 10% of samples presenting with clinical disease were DENV seropositive, prior reports indicate this is likely an underestimate of DENV travel exposure. Analysis of returned travellers from DENV-endemic countries in Boston demonstrates 12% DENV antibody positivity, with 85% of these seropositive travellers reporting no apparent history of dengue disease [24]. Thus, with rising DENV numbers and increased frequency of multiple travels to DENV-endemic regions, the potential for a secondary infection and severe DENV in the SA returned traveller is likely to increase. This gives greater importance to ensuring local abilities to effectively recognise and manage DENV and dengue disease and the future diagnostics, including novel biomarkers to delineate the potential for severe dengue disease [12,34].

A DENV NS1/IgM/IgG rapid immunochromographic test is used as a diagnostic in SA, since it is a convenient, quick turn-around test to utilise in the setting of low demand. This test, however, is only ‘laboratory suggestive evidence’ of probable DENV infection, in accordance with the Australian Government Department of Health dengue virus infection case definition (http://www.health.gov.au/internet/main/publishing.nsf/content/cda-surveil-nndss-casedefs-cd_dengue.htm). Here we assessed the utility of RT-PCR as a diagnostic, which would bring laboratory evidence in line with the notifiable diseases requirements for ‘confirmed’ dengue. Using an RT-PCR approach to detect all DENV strains (pan-DENV) and published primers [15], only 43.8% of samples returned DENV-positive results. This was not due to the lack of DENV RNA in sera, since additional testing using published serotype-specific primers [17] increased the RT-PCR positivity rate to 87.5%.

Consistent with the variable results for RT-PCR positivity and prior genotypic analysis of DENV in the returned traveller to Queensland [28], specific serotype RT-PCR analysis highlighted diversity in the DENV strains in this cohort. Results demonstrate the predominance of DENV-2, followed by DENV-1, as reported to circulate in Indonesia in 2014/15 [35], the major travel destination of this cohort. Since Indonesia and Thailand are primary travel destinations where dengue was acquired in our cohort, and these countries have been suggested as sources via air travel for movement of different DENV serotypes throughout SEA [36], the recent reports of DENV-3 predominance in regions of Indonesia [37,38] suggest that this serotype is likely to become more common in the DENV-infected traveller returning to SA. RT-PCR suggested the potential for mixed infections, with serotype-specific positivity observed for DENV-1/-2 and DENV-2/3. Mixed co-infections with more than one DENV serotype have been reported [39,40], including in DENV-endemic countries such as Indonesia [41] and in our own prior study in Sri Lanka (Senaratne et al., manuscript submitted). In the study here, however, cross-reactivity at approximately hundredfold lower level was observed with the Mon601 DENV-2 used as a negative control in the DENV-1 serotype-specific RT-PCR assay, suggesting that a DENV-2 strain can be detected in the DENV-1 assay under some conditions. Therefore, results here recording DENV-1/-2 RT-PCR-positivity cannot be conclusively deemed a mixed DENV serotype infection. Overall, the use of a RT-PCR diagnostically in this setting is complex and will require assays with multiple DENV targets and primer sets, but may be useful in a combination approach with existing NS1 antigen and serological tests, as previously suggested [12]. While this would provide additional information on DENV strains in SA and potentially useful information to reflect future risk of a second serotype infection and severe disease, this is unlikely to be a cost-effective, simple addition to present routine DENV diagnostics in SA.

Since the start of this project and collection of patient samples, ZIKV has emerged on the global stage [42], which can complicate the clinical and diagnostic picture for DENV [7,18,43]. RT-PCR analysis was also performed for ZIKV on these patient samples, with no positive samples identified. While it is now clear that urine is a better diagnostic sample for confirmation of ZIKV, and there have been case reports of ZIKV in Indonesia [44], a study during the 2014/15 DENV outbreak in Jambi, Indonesia, identified only one case of ZIKV infection out of 103 DENV cases [45]. This supports the rarity of ZIKV in SEA and is consistent with our failure to identify ZIKV infection in our 2014/15 returned traveller cohort.

## 5. Conclusions

In conclusion, this study establishes the common clinical and laboratory profiles of travellers returning to SA with DENV infections. DENV-positive results were seen in the febrile returned traveller from SEA, with locally-acquired circulating strains (DENV-1 or -2) and presenting with rash, laboratory features of neutropenia, leukopenia, thrombocytopenia and relatively low CRP for febrile illness. Convalescence sera within 3 weeks of illness and DENV RT-PCR at presentation added little further clinically-useful information. Although there is no specific treatment for dengue, it is important that doctors be able to recognise cases of DENV infection and identify dengue warning signs in order to effectively manage patients, with the potential for the incidence of dengue and severe dengue associated with diverse DENV serotypes, likely to increase in SA in the future.

## Figures and Tables

**Table 1:** Primers used for pan-dengue virus (DENV), DENV serotype-specific and Zika virus (ZIKV) RT-PCR. Y = C/T; K = G/T; W = A/T.

RT-PCR		Sequence	Ref.
Pan-DENV	Forward	AAGGACTAGAGGTTAKAGGAGACCC	
	Reverse	CGYTCTGTGCCTGGAWTGATG	[15]
	Probe	FAM-TCTGGTCTTTCCAGCGTCAATATGCTGTT-BHQ	
DENV-1	Forward	GACACCACACCCTTTGGACAA	[17]
	Reverse	CACCTGGCTGCTACCTCCAT	
DENV-2	Forward	GACCACACGYAACGGAGAA	[17]
	Reverse	TCTGTTTTRAACAGRAGACTTTT	
DENV-3	Forward	GGGAARACCGTCTATCAATA	[17]
	Reverse	CGCCATAACCAAYTTCATTGG	
DENV-4	Forward	TGAAGAGATTCTCAACCGGAC	[17]
	Reverse	AATCCCTGCTGTTGGTGGG	
ZIKV	Forward	CCTTGGATTCTTGAACGAGGA	[18]
	Reverse	AGAGCTTCATTCTCCAGATCAA	

**Table 2:** Summary of DENV serological and RT-PCR testing in patients who were subsequently diagnosed with DENV (*n* = 49, serology; *n* = 32, RT-PCR) and underwent convalescence follow-up (*n* = 5). RT-PCR results are discussed in a later section of the text.

Diagnostic Parameter	# Number (%) Seropositive		RT-PCR		
			# of samples	Pan-DENV PCR positive	Positive by any PCR
NS1 positive	18 (37%)		12	5 (42%)	10 (83.3%)
IgM positive	9 (18%)		3	0	3 (100%)
IgM & NS1 positive	16 (33%)		12	7 (58%)	10 (83.3%)
IgM & IgG positive	3 (6%)		2	0	2 (100%)
IgG & NS1 positive	1 (2%)		1	1 (100%)	1 (100%)
IgM, IgG & NS1 positive	2 (4%)		2	1 (50%)	2 (100%)
*TOTAL* *Convalescence follow-up*	49 5 (10%)			14/32 (43.8%)	28/32 (87.5%)
*Initial result*	*Follow up result*	*Days post test*			
NS1 positive	IgM positive	6			
IgM positive	IgM positive	15			
NS1 positive	IgM positive	19			
IgM positive	IgM positive	20			
NS1 positive	IgG & IgM	22			

**Table 3:** Demographic characteristics of the study cohort of DENV-seropositive (*n* = 49) and DENV-seronegative (*n* = 51) patients.

	DENV Seropositive	DENV Seronegative	*p*-Value
Number of Patients	49	51	
Age (Years), median (Range)	40 (27.75–51.5)	33.5 (22.75–55.5)	
Gender			
Male	24 (48.98%)	37 (60.66%)	0.2505
Female	25 (51.02%)	24 (39.34%)	
Centre of care			
Hospital	27 (55.10%)	39 (64.93%)	0.4340
Community	22 (44.90%)	22 (36.07%)	

**Table 4:** Travel histories for DENV-seropositive (*n* = 49) and DENV-seronegative patients (*n* = 51).

Travel Destination	DENV Seropositive	DENV Seronegative
Number of patients	49	51
Unrecorded travel history	12 (24.5%)	10 (19.6%)
Nil recent travel history	0	2 (3.9%)
Southeast Asia	36 (73.5%)	31 (60.8%)
Indonesia	21 (42.9%)	12 (23.5%)
Thailand	6 (12.2%)	6 (11.8%)
Vietnam	1 (2.0%)	2 (3.9%)
Singapore/Malaysia	4 (8.2%)	2 (3.9%)
Philippines	1 (2.0%)	1 (2.0%)
Borneo	1 (2.0%)	0
Cambodia	0	4 (7.8%)
Myanmar	1 (2.0%)	1 (2.0%)
Other (unspecified)	1 (2.0%)	3 (5.9%)
Australia	0	3 (5.9%)
North Queensland	0	3 (5.9%)
Pacific Islands	1 (2.0%)	5 (9.8%)
Tonga	1 (2.0%)	0
Fiji	0	3 (5.9%)
Vanuatu	0	2 (3.9%)
Indian subcontinent	3 (6.1%)	2 (3.9%)
India	2 (4.1%)	1 (2.0%)
Pakistan	0	1 (2.0%)
Bangladesh	1 (2.0%)	0
Africa	0	2 (3.9%)
Tanzania	0	1 (2.0%)
Guinea	0	1 (2.0%)
Hong Kong	0	1 (2.0%)
Papua New Guinea	0	2 (3.9%)
Recent travel declared (destination unspecified)	0	4 (7.8%)

**Table 5:** Number of patients who presented with symptoms of dengue, as defined in the 2009 World Health Organization (WHO) guidelines, seropositive (*n* = 39) and seronegative (*n* = 51) for DENV.

Symptom	DENV Seropositive	DENV Seronegative	*p*-Value
Fever	37 (95%)	36 (71%)	0.0054
Headache	16 (41%)	16 (31%)	0.3801
Retro-orbital pain	0 (0%)	1 (2%)	1
Nausea and/or vomiting	10 (26%)	16 (31%)	0.6418
Swollen glands	0 (0%)	0 (0%)	1
Myalgia and/or arthralgia	22 (56%)	19 (37%)	0.0890
Rash	14 (36%)	4 (8%)	0.0013

**Table 6:** Laboratory or clinical diagnosis of patients that were seronegative for DENV (*n* = 51). Diagnosis is grouped and specific diagnosis indicated with *n* = 1 patients, unless specified.

Grouping	Specific Diagnosis 35/51, 68.6%
**Gastroenteritis**	Total, *n* = 5 (13.5%) *Shigella* spp. *Salmonella* spp. (*n* = 2) *Salmonella typhi* (*n* = 2)
**Respiratory**	Total, *n* = 10 (27%) Influenza A virus (*n* = 5) Pneumonia (*n* = *2*) Viral pharyngitis Parainfluenza 1 with COPD Rhinovirus with adenovirus
**Parasitic**	Total, *n* = 6 (21.6%) Malaria (*n* = 3) Cryptosporidium Hydatid disease Giardia
**Other**	Total, *n* = 11 (29.7%)
**Infectious**	Total, *n* = 7 Infected lymphocoele Spontaneous peritonitis *Streptococcus pyogenes* bacteremia Polymicrobial wound infection Measles virus *E coli* urosepsis Infective endocarditis Febrile or ‘viral’ illness (*n* = 3)
**Non-infectious**	Total *n* = 4 Haemophagocytic lymphohistocytosis Phototoxic rash Gastric diffuse large B-cell lymphoma Benign headache
**No specific diagnosis**	Total, *n* = 16 (31.4%)

**Table 7:** Full blood examination (FBE) and inflammatory marker results for patients in DENV-seropositive (*n* = 39) and DENV-seronegative (*n* = 51) patient populations.

Laboratory Measure	Reference Range	DENV Seropositive Median (IQR)	DENV Seronegative Median (IQR)	*p*-Value
Haemoglobin				
Female	(115–155 g/L)	132.5 (128–143.25)	140 (137–148)	0.680
Male	(135–175 g/L)	149 (134–155.5)	140.5 (135.25–153)	0.316
Platelets	(150–450 ×10^9^/L)	140 (106.5–183.8)	231 (165.5–277.5)	<0.0005
Haematocrit	(0.35–0.45)	0.42 (0.39–0.43)	0.41 (0.393–0.43)	0.520
White cell count	(4.00–11.0 ×10^9^/L)	2.8 (2.1–4.8)	7.41 (5.295–9.8)	<0.0005
Lymphocytes	(1.50–3.50 ×10^9^/L)	0.79 (0.54–1.32)	1.28 (0.98–2.045)	0.0218
Neutrophils	(1.80–7.50 ×10^9^/L)	1.67 (1.15–2.71)	4.91 (3.595–8.36)	<0.0005
CRP	(<8.0 mg/L)	7.25 (4.5–16)	51.5 (15.75-91.5)	<0.0005

**Table 8:** Summary of results from pan-DENV and serotype-specific DENV RT-PCR. Number of patient samples in each group are shown.

	Pan-DENV	No Serotype Determined	Mixed Serotype Result	DENV-1	DENV-2	DENV-3	DENV-4
**Pan-DENV positive**	14	4	5	1	4	0	0
**Pan-DENV negative**	18	4	2	4	7	1	0
**DENV-1**	-			5	5	0	0
**DENV-2**	-			5	11	2	0
**DENV-3**	-			0	2	1	0
**DENV-4**	-			0	0	0	0
